# Case Report: The huge esophageal displacement: a case of dramatic esophageal interfraction motion during neoadjuvant chemotherapy and immunotherapy

**DOI:** 10.3389/fimmu.2025.1602186

**Published:** 2025-07-24

**Authors:** Renba Liang, Jing Jin, Jianghu Zhang, Jun Liang

**Affiliations:** Department of Radiation Oncology, National Cancer Center/National Clinical Research Center for Cancer/Cancer Hospital & Shenzhen Hospital, Chinese Academy of Medical Sciences and Peking Union Medical College, Shenzhen, China

**Keywords:** esophageal cancer, chemotherapy, immunotherapy, motion, radiotherapy

## Abstract

**Background:**

The average movement of esophagus is about 0.15 to 0.4 cm radially and 0.3 to 0.9 cm in the superior-inferior during radiotherapy. Little study has reported giant shift of esophagus during neoadjuvant chemotherapy and immunotherapy.

**Case presentation:**

Here we presented a case of a 72-year-old male patient diagnosed with advanced lower thoracic esophageal squamous cell cancer, clinical T2N2M1, stage IVB (pulmonary metastasis). The esophagus moved about 4.2 cm radially from the right to the left of the aorta after 2 cycles of chemotherapy and immunotherapy.

**Conclusion:**

All in all, we treated a patient with advanced lower thoracic esophageal squamous cell cancer that occurred a dramatic radial escape of about 4.2 cm after 2 cycles of chemotherapy and immunotherapy.

## Introduction

Esophageal cancer, one of the most common malignancies, is ranked 7th in incidence and 6th in mortality globally in 2020 (1). According to its pathological characteristics, esophageal cancer can be divided into two types, esophageal squamous cell carcinoma (ESCC) and esophageal adenocarcinoma (EAC), which have substantial differences in behavioral characteristics, treatment and prognosis. Chemotherapy combined with immunotherapy is the standard first-line treatment for distant metastatic esophageal cancer (2, 3). However, the efficacy has reached a plateau. Recent studies have indicated that systemic therapy plus local radiotherapy can not only improve the swallowing status and improve the quality of life of patients, but also stimulate the immune response, which ultimately results in clinical benefits for patients (4–6). For resectable or potentially resectable locally advanced ESCC, the standard therapy strategy is neoadjuvant chemoradiotherapy followed by surgery, as evidenced by the 43-49% pathologic complete response rate and 60% 5-year overall survival rate in the CROSS study and NEOCRTEC5010 study (7–10). For unresectable locally advanced ESCC, definitive chemoradiotherapy is the standard treatment. Herein, we reported a patient with advanced lower thoracic esophageal squamous cell cancer whose esophagus moved about 4.2 cm radially from the right to the left of the aorta after 2 cycles of chemotherapy and immunotherapy.

## Case presentation

**Figure 1:**
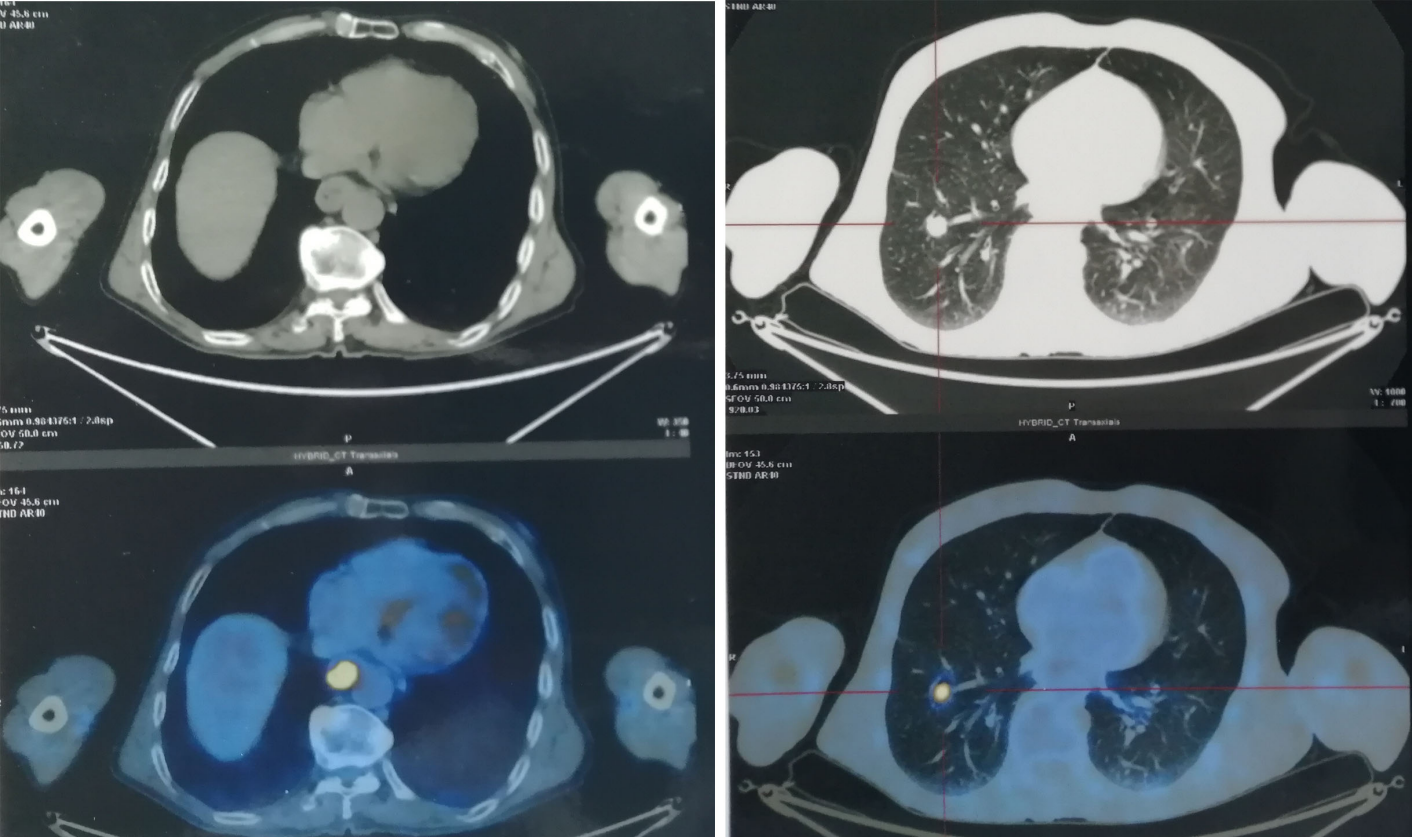
The PET/CT images showed a fludeoxyglucose avid primary mass of esophagus with pulmonary metastasis before treatment.

**Figure 2:**
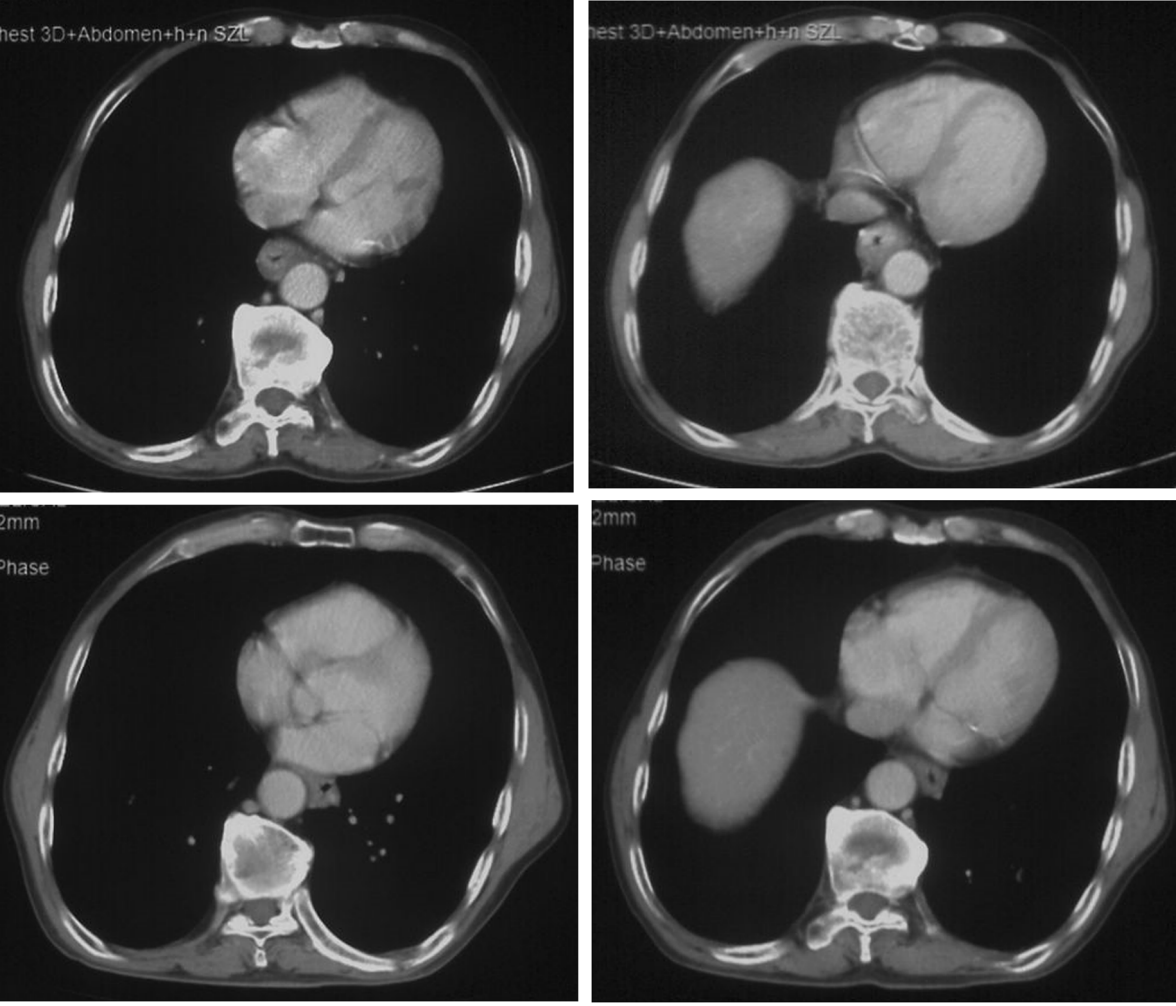
The CT images showed that esophagus had a dramatic radial escape after 2 cycles of chemotherapy and immunotherapy (lower) compared with the images before treatment (upper).

**Figure 3:**
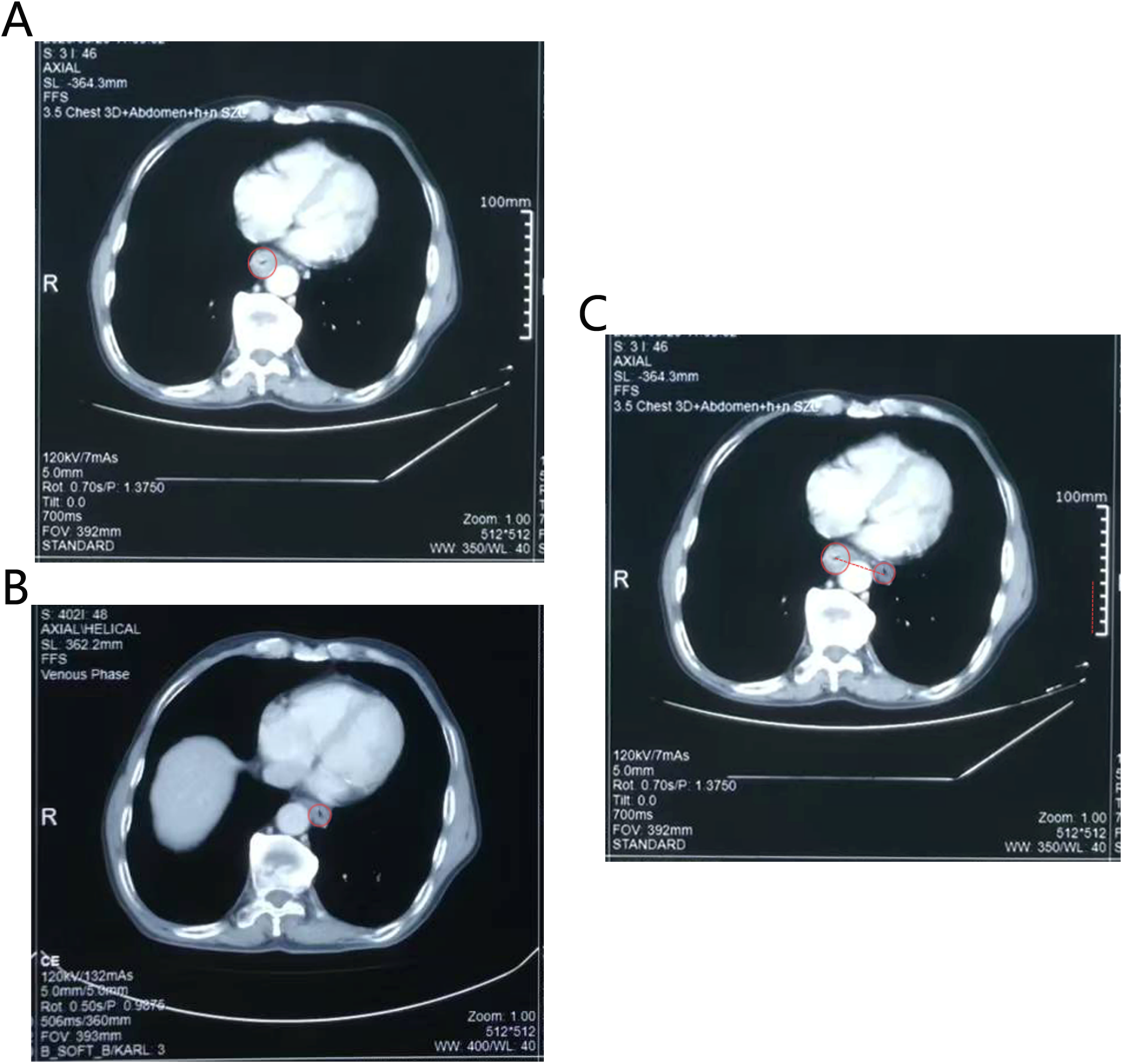
The CT images showed that esophagus had a dramatic radial escape after treatment. **(A)** The position of the esophagus before treatment. **(B)** The position of the esophagus after treatment. **(C)** The changes in the position of the esophagus before and after treatment. The red dotted line represented the distance that the esophagus moved. Technical details was in the CT images (e.g., slice thickness: 5mm).

A 72-year-old Chinese male patient was diagnosed with lower thoracic esophageal squamous cell cancer (cT2N2M1, stage IVB with pulmonary metastasis, AJCC 8th Edition) at National Cancer Center/National Clinical Research Center for Cancer/Cancer Hospital & Shenzhen Hospital, Chinese Academy of Medical Sciences and Peking Union Medical College. The esophagogastroduodenoscopy revealed a ring of 1/2 week ulcer mass extending from 34 to 39 cm from the incisors. The positron emission tomography computed tomography (PET/CT) showed a fludeoxyglucose avid primary mass with pulmonary metastasis (**Figure 1**). Therefore, the patient first received 4 cycles of chemotherapy and immunotherapy, followed by local palliative radiotherapy and immunotherapy. The chemotherapy and immunotherapy regimen were paclitaxel, cisplatin and Pembrolizumab. Interestingly, the CT revealed that malignant lesion of esophagus shifted about 4.2 cm radially from the right to the left of the aorta after 2 cycles of chemotherapy and immunotherapy. (**Figures 2**, **3**) Moreover, there was a decrease in crosssectional area of malignant lesion. (**Figure 2**). The patient continued to follow the planned treatment regimen and the esophagus never shifted back to the location seen in the staging PET/CT scan before treatment.

## Discussion

The primary first-line treatment for advanced esophageal cancer is chemotherapy plus immunotherapy (11, 12). However, as for locally advanced or node positive esophageal cancer, chemoradiotherapy is the main initial treatment strategy, with almost double the survival rate in the preoperative setting compared with surgery alone (9). Interestingly, a studies has shown that chemoradiotherapy combined with immunotherapy can improve the efficacy of patients with inoperable esophageal cancer and the toxicity is acceptable (13). Phase III studies of concurrent chemoradiotherapy combined with immunotherapy for inoperable esophageal cancer (e.g. Keynote975, Rationale311, KUNLUN et al.) are ongoing (14–16).

In light of the position near the lungs and diaphragm, the movement of esophagus is closely related to breathing, cardiac movement, and peristalsis (17–19). In addition, the esophagus shrinks in volume and tends to move from right to left during the treatment (20, 21). Studies have revealed that the average movement of the esophagus is about 0.15 to 0.4 cm radially and 0.3 to 0.9 cm in the superior-inferior during radiation therapy (20–22). However, we found an interesting phenomenon that there was a large escape of the esophagus after chemotherapy combined with immunotherapy, with a movement of about 4.2cm radially from the right to the left of the aorta in our patient. This dramatic escape was intricate and the underlying cause was still unclear. Until now, there were no specific biomarkers to explain this mechanism. Moreover, there was a pathological diagnosis before treatment, and taking pathological samples again after treatment was unacceptable to the patients and their families. Therefore, it was unknown whether there was treatment-induced fibrosis or immunotherapy-related inflammatory responses resulted in the huge movement of the esophagus. The case gives us an attention that radiation oncologists should focus on cone beam CT (CBCT) scans (once or twice a week) in order to timely discover poor target coverage of esophageal cancer when using chemoradiotherapy combined with immunotherapy.

The landmark CROSS trial had demonstrated that esophageal tumor contours were expanded with 1.5 cm radial and a 3- to 4-cm craniocaudal margin (9). Recent studies indicated that the large majority of tumor targets were covered by about 2 to 7.5 mm radially and 5 to 15 mm craniocaudally, with distal tumors requiring more distance (23). Despite the rapid development of radiation technology and machines, poor target coverage and toxicity of adjacent normal tissues is still a problem that cannot be ignored during radiotherapy. Image guidance, such as daily KV imaging or daily CBCT (once or twice a week), is an essential solution to this dilemma. Part of patients require resimulation and adaptive planning. a study suggested that radiotherapy treatment in esophageal cancer was adapted if the volume receiving 95% of the prescribed dose (V95%) coverage of CTV decreased > 1% or planning target volume (PTV) decreased by > 3% (24). However, this criteria of adaptive planning is definitely not applicable to this case, because of the huge displacement of the esophagus. Take this case for example. If the patient was diagnosed with locally advanced esophageal cancer and treated with combination of chemoradiotherapy and immunotherapy, he would benefit from twice or more a week CBCT and resimulation.

## Data Availability

The raw data supporting the conclusions of this article will be made available by the authors, without undue reservation.
